# Squalene Emulsions for Parenteral Vaccine and Drug Delivery

**DOI:** 10.3390/molecules14093286

**Published:** 2009-09-01

**Authors:** Christopher B. Fox

**Affiliations:** Infectious Disease Research Institute, 1124 Columbia St, Ste 400, Seattle, WA 98104, USA; E-Mail: cfox@idri.org

**Keywords:** squalene, squalane, adjuvant, emulsion, parenteral

## Abstract

Squalene is a linear triterpene that is extensively utilized as a principal component of parenteral emulsions for drug and vaccine delivery. In this review, the chemical structure and sources of squalene are presented. Moreover, the physicochemical and biological properties of squalene-containing emulsions are evaluated in the context of parenteral formulations. Historical and current parenteral emulsion products containing squalene or squalane are discussed. The safety of squalene-based products is also addressed. Finally, analytical techniques for characterization of squalene emulsions are examined.

## 1. Introduction to Squalene and Emulsions

Squalene is widely used for numerous vaccine and drug delivery emulsions due to its stability-enhancing effects and biocompatibility. Emulsions containing squalene facilitate solubilization, modified release, and cell uptake of drugs, adjuvants, and vaccines. Squalene and its hydrogenated form, squalane, have unique properties that are ideally suited for making stable and non-toxic nanoemulsions. Because of these characteristics, numerous squalene-based emulsions have been effectively developed for drug and vaccine applications.

**Figure 1:**
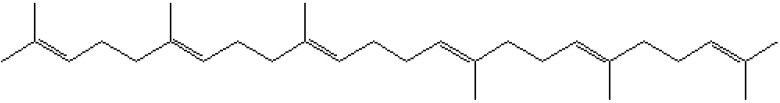
Chemical structure of squalene.

The chemical structure of squalene is that of a linear triterpene (Figure 1). The hydrocarbon composition of the molecule results in a highly hydrophobic nature; the calculated values for octanol/water partitioning coefficient (log P) and solubility of squalene in water are 10.67 and 0.124 mg/L, respectively [1]. A liquid at room temperature, squalene oil has a viscosity of ~11 cP, a surface tension of ~32 mN/m, and a density of 0.858 g/mL [2,3,4]. The X-ray crystal structure of squalene indicates a symmetric, stretched conformation [5]. The term squalene was coined in 1916 after the discovery of high concentrations of the C_30_H_50_ hydrocarbon in squaloid shark liver oil; some early reports also refer to squalene as spinacene [6,7]. Significant amounts were later found in olive oil and olive leaves [8,9,10,11,12]. In addition, squalene is present in other diverse sources such as wheat germ oil, rice bran oil, carrots, Phycomyces blakesleeanus mold, alfalfa, elderberry, and lettuce [9,13,14]. Moreover, squalene is a main component of human sebum and a precursor of cholesterol biosynthesis [6,13,14,15,16,17,18]. Interestingly, squalene from different sources has been shown to have characteristic deuterium distribution patterns, indicating varying synthesis and processing parameters [13]. The role of squalene as an important biological compound is illustrated by the fact that squalene and its related compounds oxidosqualene and bis-oxidosqualene have been discovered as precursors to almost 200 natural product triterpenoids [19]. The biosynthesis of these triterpenoids follows the biogenetic isoprene rule, a systematic reaction where the squalene precursors are catalyzed by triterpene synthases such as squalene cyclase to create a large diversity of squalene derivatives [19].

There have been concerns regarding the sustainability of obtaining squalene from sources such as sharks [20,21], not to mention the possibility of contamination or disease which is associated with animal sources in general. Cosmetic companies, for instance, have begun obtaining squalene from more renewable sources such as olives [21]. Indeed, studies indicate that squalene (and other valuable products) can be successfully extracted from olive oil processing waste [22]. Synthetic squalene has not been reported, although a synthetic polyisoprene called Syntesqual has been described [23]. Synthetic components are advantageous for vaccine and drug applications from a regulatory perspective [24]. Regulatory standards for parenteral formulations are becoming more strict and favor complete quantitative and qualitative characterization of active ingredients and excipients, extensive physicochemical analysis, and overall component purity [25]. However, the supply of natural squalene is currently relatively inexpensive and is used in most medicinal and cosmetic products.

Squalene has found use in various applications. Along with its hydrogenated analogue squalane, it is widely employed in the cosmetics industry as an emollient [13,18,26,27]. Interestingly, squalene is also used as a model compound to study vulcanization processes of natural rubber, which is also a polyisoprene [28,29]. Another novel application of a squalene emulsion employs a complex surfactant mixture that could be useful as a replacement of organic solvents used in dry-cleaning applications [30]. Beneficial physiological properties have been demonstrated by squalene, including anticancer and antioxidant activity, and it may be one of the reasons that Mediterranean diets have proven to be healthy [12,13,14,18,31]. In addition, squalene has been found to be a good marker for postprandial lipoproteinemia [32]. Because of its biocompatibility, squalene makes an attractive choice not just for cosmetics but for medicinal products as well. Thus, squalene has essentially become the *de-facto* oil of choice for parenteral vaccine emulsions and is also used for many pharmaceutical emulsions. The various reports describing the use of squalene in these parenteral formulations are reviewed below after an introduction to emulsions in general. Several references are also made to squalane, which has similarly found use in an array of medicinal and cosmetic applications [33], and is also found naturally in sebaceous secretions [34].

Emulsions are of interest in pharmaceutical and vaccine applications for several reasons. For instance, a common challenge in drug discovery is overcoming drug insolubility or instability in order to increase the bioavailability of the active compound. Emulsions can help solubilize lipophilic drugs and decrease aqueous instability by associating them with a hydrophobic oil phase [35]. In addition, emulsions offer a slower release of drug from the formulation. Moreover, since emulsions are particulate in nature, they have longer biological residence times and are more effectively phagocytosed by scavenging cells than aqueous formulations [36,37]. Thus, they can increase drug or vaccine uptake into cells. In order for an emulsion to be an effective pharmaceutical or vaccine vehicle, it is essential that the emulsion components create a stable formulation without adversely affecting the safety profile of the active compound. In addition, the physicochemical characteristics of the emulsion are important for activity. For example, smaller diameter particles (<~500 nm) can apparently travel faster to lymphatics and are more efficiently endocytosed than larger ones [36,37,38,39]. Finally, emulsions themselves have multiple adjuvant effects when added to vaccine antigens. These mechanisms of emulsion adjuvant activity are not completely understood and ongoing studies are seeking to address the issue [4,40,41,42]. 

**Figure 2:**
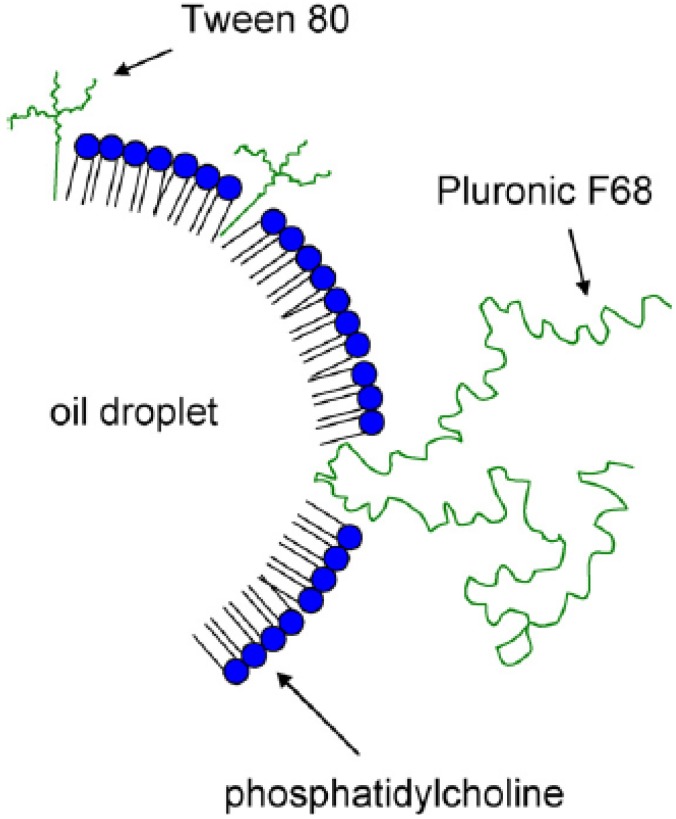
Schematic of oil droplet emulsified by various surfactants in aqueous bulk phase. Figure taken with permission from reference [76].

An immiscible oil and water mixture can be emulsified using an appropriate surfactant to create an oil-in-water (o/w) emulsion (oil droplets surrounded by aqueous bulk phase) or, conversely, a water-in-oil (w/o) emulsion (water droplets surrounded by oil bulk phase). Some emulsions are ‘self-emulsifying’ (spontaneous formation upon gentle mixing with water) while others require various levels of energy input obtained through temperature increase, blending, sonication, high-pressure homogenization (*i.e.,* microfluidization), or other methods. Droplet diameters can range from nanometers to microns and larger. The factors that determine what type of emulsion is created include the concentration of oil, water, and surfactant(s); the structures of oil and surfactant(s); temperature; and processing conditions. A schematic of an emulsified oil droplet with various emulsifiers is depicted in Figure 2. In an o/w emulsion, it is generally assumed that the oil droplet is surrounded by the emulsifying surfactants, which are in contact with the bulk aqueous phase. In general, o/w emulsions are considered more biocompatible than w/o emulsions, which are more viscous, remain longer at the site of injection, and have higher incidences of reactogenicity.

The selection of optimal surfactants is often based on the nature of the oil and whether an o/w or w/o emulsion is desired [43,44]. A scale called the hydrophilic-lypophilic balance (HLB) has been created to classify surfactant emulsifying properties. This scale ranges from 1 (lypophilic) to 20 (hydrophilic), although higher HLB values (more hydrophilic) are routinely reported as well. A surfactant with a high HLB value interacts extensively with water, whereas a low HLB value indicates a preference for oil. Oils have ‘required HLB’ values for w/o or w/o emulsions where they are optimally stabilized by emulsifiers. Moreover, combinations of surfactants have been found to create more stable emulsions, possibly due to tighter molecular packing at the oil/water interface. Thus, a surfactant with a low HLB value can be combined with a high HLB value surfactant to create a stable interfacial film. For example, the most commonly used squalene emulsion for vaccine formulations, MF59^®^, employs a 50/50 mixture of low HLB and high HLB surfactants to create an overall HLB value of 8.4. Nevertheless, a required HLB value for squalene has not been reported in the literature and the various squalene-containing emulsions described below have a wide range of HLBs. Interestingly, vaccine w/o emulsions employing squalane showed that slight variations in surfactant HLB values had a significant effect on vaccine efficacy [45]. Moreover, it has been shown that emulsion surfactants themselves can have significant biological activity [46,47,48].

Emulsion stability is a primary concern for drug and vaccine manufacturers. Instability can be caused by many factors, such as droplet flocculation or coalescence, creaming or phase separation, chemical degradation, and Ostwald ripening (a physicochemical phenomenon whereby emulsified droplets increase in size due to diffusion of molecules from smaller to larger droplets based on differences in interfacial Laplace pressure). Emulsion stability can be optimized by appropriate selection of oil, surfactants, and aqueous components as well as processing conditions. Squalene, for instance, is essentially insoluble in water. Thus, any instability due to Ostwald ripening is unlikely since the squalene molecules would be unlikely to diffuse through the aqueous medium [49]. Indeed, squalene can be used in combination with other oils to reduce their tendency for Ostwald ripening and increase emulsion stability [50]. On the other hand, the chemical structure of squalene, which includes many double bonds, may indicate the potential of chemical degradation through oxidation [31,45,51]. Thus, squalene in olive oil has been shown to undergo oxidation over time; oxidation rate increased with oxygen exposure or decrease of α-tocopherol, an antioxidant [31]. Conversely, squalene itself has demonstrated antioxidant properties, providing protection to lipids from undergoing peroxidation [14,18,31]. Therefore, stability studies are recommended to determine if degradation by oxidation is an issue in squalene emulsions and whether addition of other antioxidants or buffers for pH control is warranted [52]. Along these lines, an unbuffered version of the squalene o/w emulsion MF59^®^ experienced an unexplained loss in squalene content at 25 or 37 °C over a 3-month period [53].

Emulsions for parenteral use have additional stability and safety requirements that should be considered. For example, parenteral emulsions must be sterile, either by 0.2 μm filtration or some other means such as autoclaving. Parenteral emulsions should avoid extreme pH values and are preferably isotonic to ensure biocompatibility. Emulsion components should be regarded as generally safe for parenteral use. For instance, metabolizable oils and emulsifiers are most desirable. Parenteral emulsions employing oils other than squalene have been used extensively in the clinic for many years (e.g. Intralipid^®^).

What follows is a review of the published reports on specific squalene- or squalane-containing emulsions used in vaccine or drug formulations. Whenever possible, specific formulation components and their concentrations have been listed, with a focus on o/w emulsions. The purpose for this is to allow easy comparison between the different formulations and to make clear the presence of other excipients which may have both physicochemical and biological effects. For example, surfactants by themselves or emulsified with squalene can have significantly differing biological effects based on their structure [47,48]. Specifying exact component concentrations can be a confusing undertaking since many investigators do not specify exact compositions or do not clarify whether published compositions are diluted before injection. Many emulsions are manufactured at a certain concentration and then diluted before injection for practical reasons. Another point of confusion is that many composition concentrations are listed as a % value without specifying whether the value represents a weight/volume (w/v) or volume/volume (v/v) percentage. This review attempts to rectify these uncertainties, where possible, by specifying concentrations both at manufacture and at injection (*i.e.,* at final dilution) as well as explicitly stating % w/v or v/v values.

## 2. Vaccines

Although squalene and squalane had been used earlier without antigen to increase nonspecific immunity against tumors, it has been claimed that the first vaccine emulsion to employ squalene or squalane with an antigen was Syntex Adjuvant Formulation (SAF) in the mid-1980s [36]. However, we have found an earlier report describing the combination of a squalane emulsion with ovalbumin in 1981 [54]. In any case, SAF has been reviewed in detail elsewhere [36]. Briefly, SAF is a squalane or squalene o/w emulsion intended to reduce the toxicity of the common w/o emulsion employing mineral oil [known as Complete Freund’s Adjuvant (CFA)] while still inducing a potent cell-mediated immune response. To this end, the mineral oil of CFA was replaced with metabolizable oils, and the w/o emulsion was exchanged for an o/w emulsion so as to eliminate the tendency of the formulation to remain at the injection site, inducing reactogenicity. Several metabolizable oils were compared, among which squalene and squalane were chosen as most effective along with the surfactant Tween^®^ 80. Other additives, such as Pluronic^®^ L121 (Pluronics^®^ are polyethylene oxide-polypropylene oxide block copolymers) or a muramyl dipeptide analogue, were found to increase SAF adjuvant properties. Emulsion processing via several microfluidization cycles facilitated reduced particle size and polydispersity, increased reproducibility, and capability for sterile filtration [33]. Remarkably, the SAF emulsion (before addition of muramyl dipeptide) was stable for six years at room temperature; even freezing temperatures did not break the emulsion [33]. The final SAF composition before muramyl dipeptide addition was 5% w/v squalane, 2.5% w/v Pluronic^®^ L121, and 0.2% w/v Tween^®^ 80 in PBS at pH 7.4 [33]. These final component concentrations for injection are obtained after diluting a 2x stock upon mixing with the antigen and/or muramyl dipeptide [55,56]. Average particle size was 150-160 nm, although this included a bimodal distribution of 270 nm and 90 nm [56,57]. Squalane was finally chosen over squalene because it was presumed to be more chemically stable than squalene (no double bonds susceptible to oxidation) [33]. SAF elicited IgG2a antibodies and the cytokine IFN-γ, and other indications of a Th1-type cell mediated immune response, although many of these potent adjuvant effects are attributable to the presence of Pluronic^®^ L121 and/or the muramyl dipeptide analogue [36,58]. Interestingly enough, SAF also was found to activate the alternative complement pathway, another possible adjuvant mechanism of action [58]. Many different antigens were combined with SAF and showed good immune activity [36]. SAF induced very little muscle irritation in humans [33], but was apparently discontinued as an adjuvant product after clinical trials revealed high reactogenicity, although this was associated with the inclusion of the muramyl dipeptide analogue [36,59,60].

**Figure 3:**
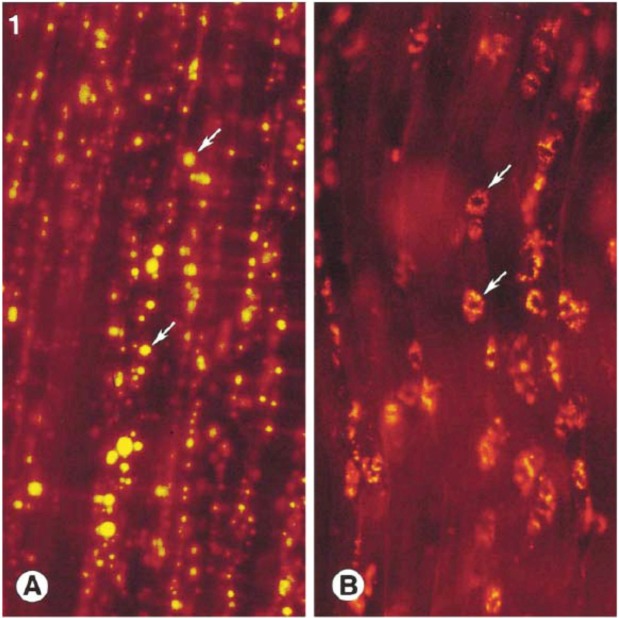
Fluorescently-labeled MF59^®^ three hours after injection has not been taken up by cells (left). At 48 hours after injection, MF59^®^ is intracellular (middle). Images taken with permission from reference [65].

Perhaps the best known squalene-based vaccine adjuvant is MF59^®^. This o/w emulsion originally included the added immunostimulant muramyl tripeptide phosphatidyl ethanolamine (MTP-PE), but this was later taken out because of toxicity [61]. MF59^®^ is manufactured as a 5% v/v squalene, 0.5% w/v Tween^®^ 80, 0.5% w/v Span^®^ 85 emulsion in 10 mM citrate buffer at pH 6, with a particle size of ~165 nm after microfluidization [53,61]. It is generally diluted 2-fold upon mixing with the vaccine antigen for injection. Interestingly, MF59 and other emulsions can be modified to include cationic emulsifiers for more effective oil-adjuvant association or cell delivery [3,62]. Although MF59 is very stable, it cannot be frozen, the squalene and surfactant components (Tween^®^ 80 and Span^®^ 85) contain unsaturated bonds that may be subject to oxidation, and pH extremes may hydrolyze Tween^®^ 80 or Span^®^ 85 [4]. MF59 has already been licensed for use in many countries as a component of the influenza vaccine Fluad^®^ [63]. It is also under investigation with several other vaccine candidates [63]. Because of its widespread use, several studies have examined MF59^®^’s adjuvant effects and mechanisms. It has been found to induce antibodies, T cell proliferation, and cyotoxic T lymphocyte activity [4]. Muscle tissue analysis after intramuscular injection of MF59^®^ in a mouse showed adjuvant-induced changes in the expression of ~900 genes (3x more than alum or CpG), including genes responsible for cytokines, cytokine receptors, leukocyte migration, and antigen presentation [40]. A study employing extensive *in vitro* cell assays concluded that MF59^®^ may increase immune cell migration to injection site, promote DC maturation and antigen uptake, and enhance DC migration to lymph nodes [41]. In addition, a different study proposed that emulsion adjuvants containing squalene such as MF59^®^ and Hjorth adjuvant (see below) enhance antigen presenting cell survival or proliferation [42]. Another report found that four hours after intramuscular injection in mice, 86% of the injected MF59^®^ was still in the muscle or surrounding fat tissue and had a half-life in mouse muscle of 42 hours [64]. This same study reported that MF59^®^ in the lymph nodes was detected as ~0.2% of the injected dose and peaked at two days after injection and that an associated antigen was cleared independently and more rapidly than the MF59^®^, meaning no antigen depot effect [64]. However, fluorescence microscopy images showed that MF59^®^ significantly increased antigen uptake into antigen presenting cells, a finding correlated by increased antibody titers of MF59^®^ associated antigens [65]. While at three hours after injection most of the MF59^®^ remained extracellular, at 48 hours most of the MF59^®^ at the site of injection had been taken up by dendritic cells (see Figure 3) or transported with antigen presenting cells to the lymph node [65].

**Figure 4:**
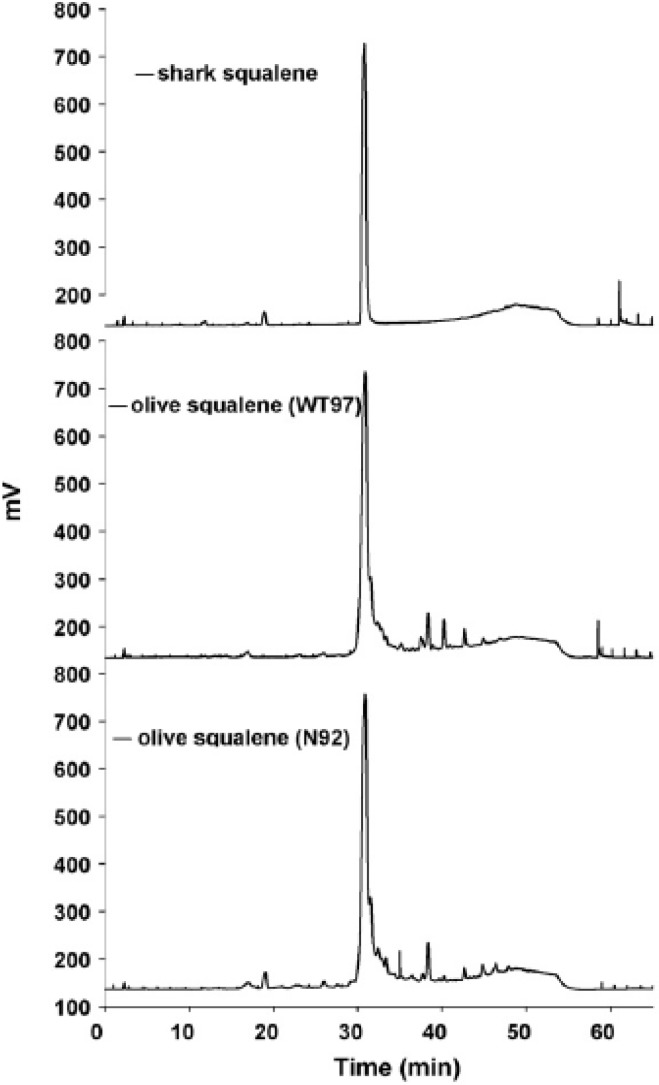
HPLC analysis with charged aerosol detection of squalene and impurities from shark or olive sources. Figure taken with permission from reference [76].

Ribi ImmunoChem Research (later acquired by Corixa, then GlaxoSmithKline Biologicals) developed several squalene or squalane-containing emulsions for delivery of immunostimulants [66]. The formulation known as DETOX^®^ includes bacterial cell wall skeleton (CWS) and monophosphoryl lipid A (MPL) in a squalane (1%), and Tween^®^ 80 (0.2%) formulation [66]. DETOX^®^ shows good adjuvant activity, but also reactogenicity and granulomas at the injection site [66,67]. Nevertheless, DETOX^®^ has been approved in a licensed therapeutic vaccine called Melacine^®^ for treatment of melanoma [68,69]. Ribi also published work on several other adjuvant emulsions, including a 10% v/v squalene, 1.2% w/v lecithin, 0.45% v/v Tween^®^ 80 mixture in water, which is diluted 5x upon injection [37,70]. The closely related and widely used formulation known as Ribi Adjuvant System (RAS) contains 2% v/v squalene, 0.2% Tween 80, and added immunostimulants such as synthetic trehalose dicorynomycolate, bacterial cell wall skeleton, and MPL. [71,72] This is now available from Sigma-Aldrich as Sigma Adjuvant System^®^ (product #S6322), consisting of 2% v/v squalene, 0.2% Tween^®^ 80, synthetic trehalose dicorynomycolate, and MPL. A similar formulation termed SE (stable emulsion) has been patented by Ribi and consists of 10% v/v squalene, 1.9% w/v lecithin, 0.091% w/v Pluronic^®^ F68, 0.05% w/v α-tocopherol, and 1.8% v/v glycerol in 25 mM ammonium phosphate buffer pH 5.1 [73,74]. Adding MPL to SE creates MPL-SE, a potent adjuvant currently in clinical trials as a Leishmaniasis vaccine [75]. Development work on SE and other adjuvant emulsions has continued at the Infectious Disease Research Institute (IDRI) [75,76,77]. IDRI has studied the physicochemical and biological effects of substituting components of different source and structure in the SE formulation, such as replacing shark squalene with olive squalene as well as comparing squalene source purity (see Figure 4) [76].

GlaxoSmithKline Biologicals has developed several squalene emulsion formulations as vaccine adjuvants. SB62 consists of 5% v/v squalene, 5% v/v α-tocopherol, and 1.8% v/v Tween^®^ 80 in PBS at pH 6.8, with a particle size of ~150-155 nm [78]. When diluted two-fold for injection, the above formulation is called AS03 [78]. AS03 has been approved as a component of the pandemic flu vaccine Prepandrix [75]. Several variations of AS03 have been reported, the most well-known being AS02. AS02 is identical to AS03 with the addition of immunostimulants MPL and QS21 [79,80,81]. Another possible variation on AS03 is to include CpG and a saponin (such as QS21) [82]. AS02 is in clinical trials for various vaccine applications, including malaria, hepatitis B, human papilloma virus, tuberculosis, and HIV [75], although some reactogenicity has been reported [83].

Sanofi Pasteur has developed a promising squalene-based o/w emulsion known as AF03 that is manufactured by cooling a pre-heated w/o emulsion until it crosses the emulsion phase inversion temperature, creating an o/w emulsion (*i.e.,* thermoreversible) [84,85,86]. This emulsion optionally contains a TLR4 agonist molecule and mannitol, and can reportedly be lyophilized. Upon injection, it consists of 2.5% w/v squalene, ~0.48% w/v Ceteareth-12, ~0.37% w/v sorbitan monooleate (also known as Span^®^ 80), and phosphate buffered saline or citrate buffer, with droplet size <100 nm.

Experimental squalene adjuvant (ESA) is composed, upon injection, of 5% w/v squalene, 4% w/v Pluronic^®^ L35, and 2% w/v of the surfactant Abil^®^-Care (polysiloxan polymer dimethicone copolyol), with a particle size of 200 nm [87]. The antigen is emulsified with the emulsion under mild conditions. This formulation was found to be more effective at eliciting immunogenicity than alum with a rabies or parvovirus vaccine. ESA exhibited a promising safety profile in guinea pigs, with low reactogenicity, low pyrogenicity levels, and no adverse sensitization [88].

Several more squalene-based emulsions for vaccines or drugs have been described in the literature, including several patents [36,89]. Various squalene emulsions have also been described for the delivery of human chorionic gonadotropin for HIV, cancer, or fertility treatments [90,91]. The combination of a squalene emulsion with polymeric particles in a tetanus toxoid vaccine significantly increased antibody titers to tetanus toxoid [92]. Mixing a squalene emulsion with alum has also been reported [93].

Water-in-oil emulsions containing squalene have also garnered interest. A widely used and effective squalene-containing w/o formulation is Montanide^®^ ISA 720, which combines 70% v/v squalene with a mannide monooleate emulsifier [4,94,95]. Although much less toxic than mineral oil adjuvants, the reactogenicity of Montanide^®^ ISA 720 may be cause for concern and is currently under investigation [83,96,97,98]. Another w/o squalene emulsion is TiterMax^®^ Classic, which contains squalene, Tween^®^ 80, the block copolymer CRL-8941 (Pluronic^®^ L141), and silica microparticles coated with CRL-8941 [87,99,100,101]. Generally, a 50% v/v squalene content is injected but this can be reduced to as low as 10% [101]. TiterMax^®^ Gold is closely related to TiterMax^®^ Classic, but employs a different polyethylene oxide-polypropylene oxide block copolymer (CRL-8300) and no silica microparticles [99,102]. TiterMax^®^ is a potent adjuvant designed to elicit similar response as CFA in research animals with reduced toxicity [99,100]. It has been compared to MF59^®^ and alum in a meningitis vaccine in mice [103]. In addition, an adjuvant formulation consisting of 35% v/v squalene emulsion emulsified with 15% v/v Arlacel^®^ A (mannide monooleate) mixed with a polycationic polyelectrolyte has been described [104]. Also of interest, a water-in-oil-in-water (w/o/w) squalene adjuvant was found to be safe and effective in protecting chickens against Newcastle disease virus [105]. It is composed of liquid paraffin (25%), squalene (10%), diglyceryl monooleate (5%), and Tween^®^ 80 (2%) in PBS.

Not surprisingly, the hydrogenated form of squalene, squalane, has also been widely applied in vaccine emulsion formulations. As mentioned above, an early report of a squalane/Tween^®^ emulsion containing muramyl dipeptide and ovalbumin described the resulting induction of a cell mediated immune response [54]. Later, SAF and DETOX^®^ employed squalane instead of squalene. A different squalane-based o/w emulsion with Tween^®^ 80 and a sulfolipo-cyclodextrin adjuvant found efficacy in veterinary applications, producing similar antibody titers as w/o formulations and an earlier and stronger cell mediated response with no reactogenicity in cattle [106]. Earlier, this adjuvant had shown efficacy eliciting high antibody titers compared to mineral oil, hexadecane, or soya oil emulsions in pigs and mice, although some reactogenicity was apparent due to the sulfolipid-cyclodextrin [107]. The immunostimulant molecular structure has since been refined and the formulation is now called CoVaccine HT, consisting (upon injection) of 8% w/v squalane, 2% w/v Tween^®^ 80, and 2% w/v immunostimulant (sucrose fatty acid sulfate ester) in PBS and was found effective in eliciting humoral and cellular responses in pigs [68,108,109]. CoVaccine HT in a therapeutic hypertension vaccine is in phase II clinical trials [110].

Other squalane formulations have also been reported. A squalane emulsion known as AF, SPT, or PROVAX^®^ has been created by IDEC Pharmaceuticals. This emulsion formulation is similar to SAF, but reduces the concentration of Pluronic L121 to 1.25% w/v upon injection and eliminates the muramyl dipeptide analogue. PROVAX^®^ is prepared as a 3x concentrate and contains 15% w/v squalane, 3.75% w/v Pluronic^®^ L121, and 0.6% w/v Tween^®^ 80. It induces both antibodies and cytotoxic T cells [36,111]. Also of interest, a w/o/w squalane-based emulsion effectively elicited IgG and IgA antibody responses to ovalbumin after oral administration [112]. 

The preferred choice of oil between squalane or squalene is not obvious. While both oils have reduced tissue reaction compared to historical mineral oils [36,45,113], squalane may be slightly more reactogenic at injection sites than squalene [37]. This may in part be due to squalane’s higher viscosity [45]. Moreover, differences in metabolizability of the two oils may play a role. As a precursor of cholesterol synthesis, it is agreed that squalene is metabolizable. However, there are conflicting opinions regarding the metabolism of squalane; its saturated chemical structure indicates that it may be more difficult to metabolize than unsaturated squalene [36,87,106,113,114]. In the case of orally ingested formulations, it is thought that non-metabolizable oils prevent effective drug bioabsorption [115]. Some consider squalane a better choice than squalene for stability purposes (*i.e.,* no double bonds subject to oxidation) [106], although this does not appear to be a serious issue with squalene since emulsions such as MF59^®^ are stable for years [63]. On the other hand, olive oil squalene clearly undergoes oxidation over time, especially with increased vial headspace (*i.e.,* more exposure to oxygen) [31]. Differences in biological efficacy of squalane and squalene are also unclear. For instance, squalene and squalane (but not peanut oil) are effective substitutes for mineral oil as bacterial cell wall carriers for antitumor activity [113,114,116]. However, in some early reports of anticancer formulations containing cell wall materials, squalane emulsions out-performed squalene emulsions in inducing tumor regression [113,117]. Similarly, w/o emulsions made of squalane showed higher HI titers than those employing squalene in a bird Newcastle disease vaccine model [45]. However, it was also shown that surfactant concentration (Tween^®^ 80) affected anticancer activity of squalene emulsions and could be optimized to produce similar efficacy as squalane or mineral oil [113]. This finding illustrates the importance of considering all formulation components and physicochemical aspects when comparing emulsions.

As indicated above, various surfactants alone or in combination with squalane have differing biological effects based on their structure and HLB value [46,47,48]. In squalene-DOTAP emulsions used for gene transfection, it was shown that various non-ionic surfactants had differing effects on transfection efficiency according to the polyethylene glycol content (Tween^®^ 80 had low toxicity and good efficiency) [118]. In a drug delivery application, various surfactants slowed drug release from a squalene emulsion at different rates [35]. A related study indicated that the surfactants Brij 30 and Brij 98 shielded the hemolytic activity of squalene-lecithin emulsions, while all surfactants affected particle size and drug release rates [119]. Moreover, studies show a complex interplay between particle size, surfactant concentration, and immunostimulant component in mineral oil emulsions for immunotherapy [113,120]. Particle size itself is generally thought to have significant effects on biological activity of particulate formulations [36,37,38]. For example, a microfluidized version of SAF (smaller droplet size) was more effective in a Hepatitis B model but showed no difference with ovalbumin compared to a non-microfluidized SAF [56,121]. Finally, although the preferred choice between squalene and squalane has not been established, it does appear that these oils have advantages over many other oils, whether from mineral or vegetable sources. As mentioned above, nutritional supplement emulsions for intravenous injection have successfully employed various oils other than squalene with lipid emulsifiers. However, the high surface tension of squalene allows it to create smaller size emulsions when emulsified with egg phosphatidylcholine than at least 17 other oils, including mineral oil, coconut oil, sesame oil, or soybean oil (see Table 1) [3,119]. As mentioned above, several investigators have found that squalene or squalane emulsions outperformed vegetable oils in vaccine adjuvant applications [107,113,114].

## 3. Drug Delivery

**Figure 5:**
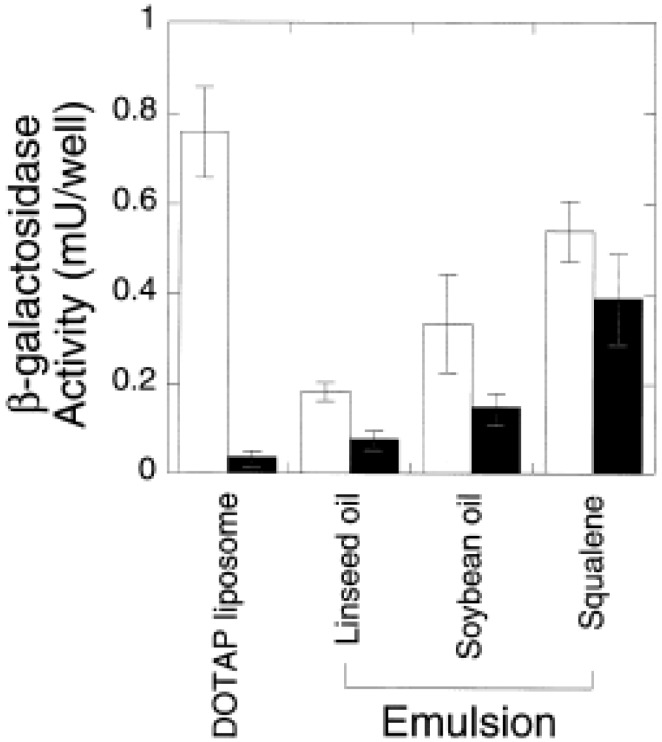
*In vitro* gene activity in the absence (open bar) and presence (closed bar) of serum as measured by β-galactoxidase activity. Figure taken with permission from reference [3].

While squalene emulsions have taken precedence in the vaccine field, there is also significant interest in pharmaceutical applications. Indeed, many of the studies cited above comparing oils and surfactants were undertaken in the drug delivery context. While squalene is effective for vaccine adjuvant applications and for making stable emulsions, the use of squalene vs. other oils for drug delivery will depend upon solubility and release characteristics of the drug in the oil. Thus, squalene-DOTAP emulsions for gene transfection had smaller particle size and more stability compared to soybean oil-DOTAP or linseed oil-DOTAP emulsions, and were more effective at *in vitro* and *in vivo* transfection efficiency than these emulsions or DOTAP liposomes while also being the least cytotoxic (see Figure 5) [3,122]. Squalane emulsions (10% v/v) with varying concentrations of DOTAP and cholesterol or DOPE as coemulsifiers were also effective gene transfection vehicles [123]. A related study showed that a 10% w/v squalene-2.4% w/v DOTAP emulsion had better *in vivo* transfection efficiency and minimal toxicity compared to liposome or poly(ethyleneimine) vehicles [124]. Furthermore, 10% v/v squalene emulsions with varying concentrations of lecithin showed good stability and the slowest release of the lipophilic drug rifampicin compared to soybean and linseed oil emulsions [3]. It should be noted, however, that *in vitro* drug release assays are not necessarily indicative of the *in vivo* situation [125]. Especially with submicron emulsions, slowed *in vivo* drug release is more likely to be achieved with drugs that have high log P values [126].

Submicron squalene-phosphatidylethanolamine emulsions slowed morphine and morphine prodrug release and induced successful anesthesia; addition of Pluronic^®^ F68 and cholesterol further slowed drug release and increased anesthesia duration [35]. An emulsion formulation in this case was especially useful since the drugs themselves were susceptible to hydrolysis in aqueous solution. A similar 10% v/v squalene, 3% w/v lecithin emulsion with 200 nm particle size and various coemulsifiers was used to deliver a morphine-like drug, nalbuphine [119]. These emulsions slowed drug release, especially in the presence of Brij^®^ 98, significantly increasing the duration and potency of analgesia. Further more, Brij^®^ 30 and Brij^®^ 98 shielded the hemolytic activity of the emulsions.

**Table 1:** Comparison of physicochemical properties of emulsions made with different oils, taken with permission from reference [3]. Surface tension and viscosity values were measured at 22 ± 2 °C and 20 ± 2 °C, respectively.

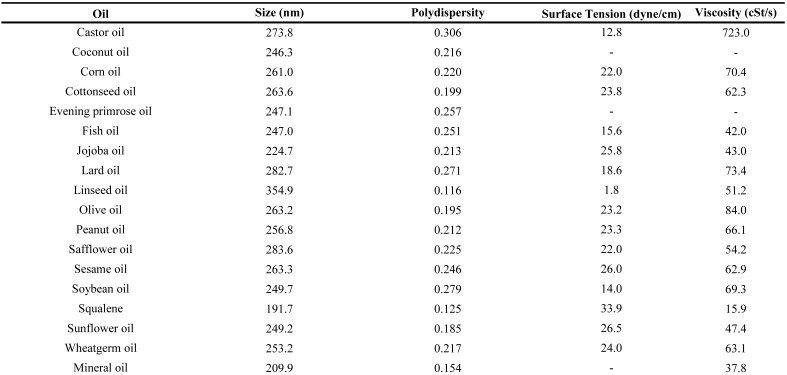

Cancer drug applications have also been reported. For instance, squalene itself is of interest as a drug because it has been shown to have chemopreventive activity [12]. In a study of an anticancer drug, camptothecin, which is highly insoluble, chemically unstable, and toxic when not formulated, lipid nanoparticles (including one containing squalene) and a 10% w/v squalene-0.2% w/v Myverol™-2.4% w/v Pluronic^®^ F68 emulsion were compared [127]. In this case, a non-squalene lipid nanoparticle was found to have the best stability, drug release, and biological activity properties while maintaining low levels of hemolytic activity. An i.v. emulsion for the administration of taxol (an insoluble cancer drug) consisting of squalane or squalene, sucrose, and emulsifiers such as Pluronic^®^ F86 and Tween^®^ 80 has also been described [128].

Finally, other research suggests that squalene in combination with prevastatin is an effective treatment for hypercholesterolemia with a low level of side effects [129]. In a related application, it was found that squalene oil was more effective than canola oil or triolein oil, but not seal oil, at loading and delivering a radioactive marker to acylated human low density lipoprotein [130]. Interestingly, i.v.-injected squalene has longer half-life than plant sterols or triglycerides [131].

To summarize the above discussion of vaccine and drug delivery emulsions employing squalene (or squalane) and to allow direct comparison between formulations, the emulsion compositions have been collated in Table 2, Table 3, Table 4. Table 2 presents squalene-based o/w emulsions, Table 3 includes squalane-based o/w emulsions, and Table 4 covers squalene-based w/o emulsions.

**Table 2:** Composition of squalene oil-in-water parenteral emulsions.

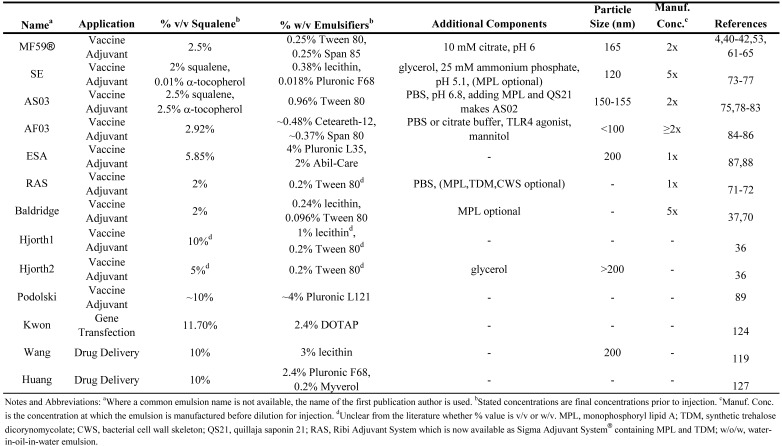

**Table 3:** Composition of squalane oil-in-water parenteral emulsions.

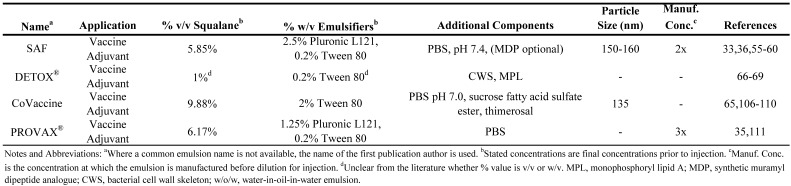

**Table 4:** Composition of squalene water-in-oil parenteral emulsions.

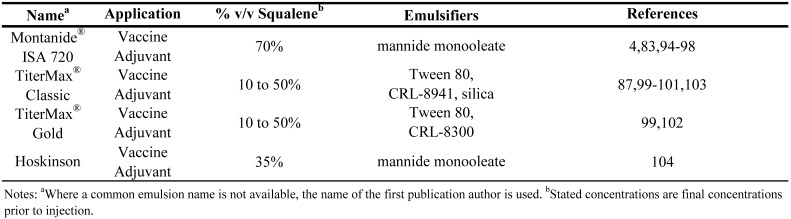

## 4. Safety

It is important to verify the safety of squalene for use in drug and vaccine products, especially in prophylactic populations and/or young children. Much has been published regarding the safety of squalene, including a concise but thorough review from the Institute of Medicine [132]. In general, squalene has an excellent safety profile: it is nonirritating, nonallergenic, poorly absorbed through the gastrointestinal tract, slowly absorbed through the skin, and has low toxicity by all routes, with an oral LD_50_ of 5 g/kg and an i.v. LD_50_ of 1.8g/kg [4,6,26,133]. Intravenous injection of 21 mg of squalene into humans together with the emulsion Intralipid^®^ induced no side effects [32]. Encouragingly, vaccine applications would typically require very little total squalene, since 1 to 2.5% v/v final oil concentration has similar adjuvanticity but minimal reactogenicity than higher squalene concentrations [37,53]. As mentioned, squalene emulsion injection sites have reduced tissue reaction compared to mineral oil emulsions, with faster healing and smaller scarring [36,45,113]. Also previously described, squalene in combination with prevastatin as a treatment for hypercholesterolemia exhibited low side effects [129]. Perhaps the strongest case for the safety of squalene in a vaccine setting is the well-documented safety record of MF59^®^, which has been reviewed elsewhere [63]. Approximately 27 million doses of MF59^®^ have been injected into humans of all age groups (including infants) with little or no adverse side effects. It has been licensed for use in 20 countries as a component of Fluad^®^ influenza vaccine (the first licensed adjuvant since alum). MF59^®^ has also been tested in many preclinical animal models which showed low severity inflammation and other minor, reversible reactogenicity, but was not genotoxic, teratogenic, or sensitization-inducing. Clinical data, mostly from Fluad^®^ trials and postmarket analysis, show a low/acceptable incidence rate of adverse events incident with MF59^®^ injection, the most common complaint being pain at injection site.

A controversial claim regarding the safety of squalene concerns allegations that the The Gulf War Syndrome (GWS) is typified by high squalene antibodies in anthrax vaccine recipients [103,134,135,136,137]. However, these claims were regarded as inconclusive based on several reasons, including the use of an unvalidated assay, lack of proper controls, small sample sizes, and the fact that the vaccine was found to contain no squalene [132,138,139,140]. A validated, quantitative squalene antibody assay was developed and used to show that anthrax or MF59^®^ vaccination recipients did not have higher levels of IgG or IgM squalene antibodies and that squalene antibodies occur naturally in humans [63,141,142,143,144,145].

There are studies regarding some oils, including squalene, that have been found to induce autoimmunity indications when 500 μL of the pure oil was injected intraperitoneally in mice [144,146], or 200 μL of pure oil intradermally in rats [147], or that neural damage in rats was induced after 20g/kg squalene per day for 4 days [148]. Similarly, one report suggested that excessive intake of oral squalene tablets caused lipoid pneumonia in a human patient [149]. Of course, all of these reports involve excessive amounts of non-emulsified squalene and so their relevance for administration of minute amounts of emulsified squalene such as would be injected in a vaccine is questionable. In summary, there is significant evidence that squalene vaccine emulsions such as MF59^®^ have an excellent safety record. Any indication of squalene toxicity at low doses is inconclusive.

## 5. Squalene and Emulsion Characterization

It has been pointed out that appropriate quantification of squalene in vaccine or pharmaceutical formulations is essential for manufacturing quality control and regulatory considerations [25,150]. Detection or characterization of squalene is possible using various analytical methods [150]. For example, squalene can be quantified by RP-HPLC or HPLC-SEC with UV, light scattering, or refractive index detection [10,28,135,151,152]. Moreover, a charged aerosol detector (Corona^®^ CAD^®^) with HPLC has been used to effectively detect squalene (see Figure 4) [76]. In fact, this technique demonstrated a lower limit of detection (<0.2 ng) than evaporative light scattering detection or atmospheric pressure chemical ionization mass spectrometry [152]. Thin-layer chromatography with fluorescence, flame ionization, or radioactive detection has also been demonstrated [15,17,153]. Gas chromatography, especially in combination with mass spectroscopy, has also been shown to be an effective squalene detection method [9,11,154,155]. Finally, NMR spectroscopy and vibrational spectroscopy are also useful, especially for structural characterization [16,29]. Indeed, site-specific natural isotope fractionation measured by deuterium NMR (SNIF-NMR) was able to differentiate squalene from different origins based on deuterium distribution [13].

**Figure 6:**
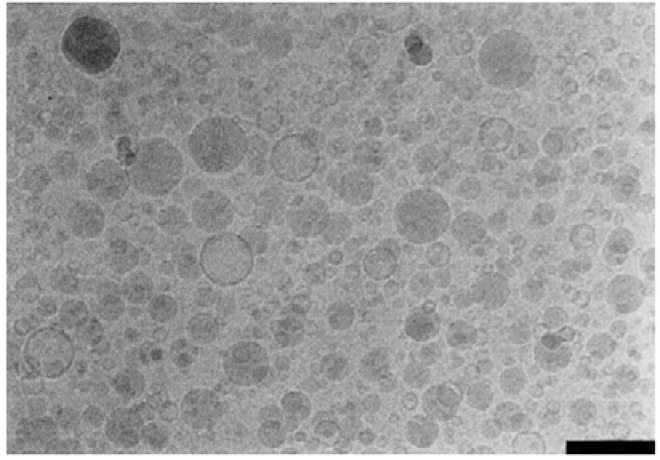
Cryo-EM image of triglyceride oil emulsion showing oil droplets (shaded) and liposomes (clear). Scale bar is 200 nm. Figure taken with permission from reference [159].

Multiple methods are available for analysis of interactions between emulsion components. There are several analytical techniques that can determine component concentration, particle size, charge, and other interfacial properties, as well as help elucidate mechanisms of emulsion instability, active ingredient association, and biological activity. A recent review [156] highlights several techniques useful for monitoring emulsion stability including visual inspection or optical profiling for phase separation, accelerated destabilization caused by high temperature or centrifugation, particle size measurements (often using dynamic light scattering [3,49,76,123]), morphological characterization using optical or electron microscopy, particle charge (zeta potential) obtained using microelectro-phoresis [123], and viscometers and rheometers for rheology characterization [3,157]. It should be noted that an additional particle sizing technique (besides dynamic light scattering) such as single particle optical sensing is necessary for determining the amount of large emulsion droplets (e.g. >5 μm) in a formulation [158]. Detection of large-sized droplets is important for safety and stability monitoring [158]. Cryo-TEM is the preferred electron microscopy method for nanoemulsions since it avoids artifacts inherent with other electron microscopy sample preparation techniques and allows clear differentiation between emulsion droplets and other structures (see Figure 6) [159]. NMR spectroscopy, differential scanning calorimetry (DSC), and surface tension measurement can be very useful to elucidate interfacial interactions [3,159]. Finally, *in vitro* or *in vivo* assays help correlate physicochemical parameters with biological effects [3,76,123].

## 6. Conclusions

In summary, squalene has proven effective for numerous vaccine and drug delivery applications due to its unique properties, including high surface tension (allowing small droplet size emulsions), stability, and biocompatibility. Squalene and squalane emulsions allow the solubilization and slowed release of lipophilic drugs, adjuvants, and vaccines, facilitating increased bioavailability and sustained mechanisms of action. Moreover, squalene emulsions demonstrate adjuvant activity such as eliciting increased antibody titers and cell mediated responses in vaccine applications. Finally, there are various established methods for the analysis of squalene and emulsion formulations, allowing thorough physicochemical characterization and stability monitoring. It is expected that squalene and squalane will continue to play a significant role as components in future vaccine and drug formulations.

## List of Abbreviations

### Abbreviations

CAD: charged aerosol detector
CpG: oligonucleotide adjuvant containing cytosine-phosphate-guanine sequences
CWS: bacterial cell wall skeleton
DSC: differential scanning calorimetry
ESA: Experimental Squalene Adjuvant
FCA: Freund’s Complete Adjuvant
GWS: Gulf War Syndrome
HLB: hydrophilic-lipophilic balance
IFN-γ: interferon-γ
MPL: monophosphoryl lipid A
MTP-PE: muramyl tripeptide phosphatidyl ethanolamine
QS21: quillaja saponin 21
RAS: Ribi Adjuvant System
SAF: Syntex Adjuvant Formulation
SE: stable emulsion
